# Higher Thermal Acclimation Potential of Respiration but Not Photosynthesis in Two Alpine *Picea* Taxa in Contrast to Two Lowland Congeners

**DOI:** 10.1371/journal.pone.0123248

**Published:** 2015-04-13

**Authors:** Xiao Wei Zhang, Jing Ru Wang, Ming Fei Ji, Richard Ian Milne, Ming Hao Wang, Jian-Quan Liu, Sheng Shi, Shu-Li Yang, Chang-Ming Zhao

**Affiliations:** 1 State Key Laboratory of Grassland Agro-Ecosystems, School of Life Sciences, Lanzhou University, Lanzhou, 730000, China; 2 Institute of Molecular Plant Sciences, The University of Edinburgh, Daniel Rutherford Building, King’s Buildings, Mayfield Road, Edinburgh, EH93JH, United Kingdom; Wageningen University, NETHERLANDS

## Abstract

The members of the genus *Picea* form a dominant component in many alpine and boreal forests which are the major sink for atmospheric CO_2_. However, little is known about the growth response and acclimation of CO_2_ exchange characteristics to high temperature stress in *Picea* taxa from different altitudes. Gas exchange parameters and growth characteristics were recorded from four year old seedlings of two alpine (*Picea likiangensis* vars. *rubescens* and *linzhiensis*) and two lowland (*P*. *koraiensis* and *P*. *meyeri*) taxa. Seedlings were grown at moderate (25°C/15°C) and high (35°C/25°C) day/night temperatures, for four months. The approximated biomass increment (ΔD^2^H) for all taxa decreased under high temperature stress, associated with decreased photosynthesis and increased respiration. However, the two alpine taxa exhibited lower photosynthetic acclimation and higher respiratory acclimation than either lowland taxon. Moreover, higher leaf dry mass per unit area (LMA) and leaf nitrogen content per unit area (N_area_), and a smaller change in the nitrogen use efficiency of photosynthesis (PNUE) for lowland taxa indicated that these maintained higher homeostasis of photosynthesis than alpine taxa. The higher respiration rates produced more energy for repair and maintenance biomass, especially for higher photosynthetic activity for lowland taxa, which causes lower respiratory acclimation. Thus, the changes of ΔD^2^H for alpine spruces were larger than that for lowland spruces. These results indicate that long term heat stress negatively impact on the growth of *Picea* seedlings, and alpine taxa are more affected than low altitude ones by high temperature stress. Hence the altitude ranges of *Picea* taxa should be taken into account when predicting changes to carbon fluxes in warmer conditions.

## Introduction

Temperature affects almost all aspects of terrestrial carbon processes, including photosynthesis and respiration [1–4], therefore these processes will likely be profoundly affected by climate change, altering the carbon balance between the atmosphere and the biosphere [2, 5, 6]. Alpine plants plays an important role in the high altitude ecosystems of Northern Hemisphere, where store the greatest fraction of carbon stocks; nevertheless, the impact of global warming has been shown not only to rise by almost 4–10°C in high altitude areas of Northern Hemisphere, by also to increase in the frequency, duration and severity of periods with exceptionally high temperatures [7]. If elevated temperatures exceed a species’ thermal optimum for metabolism, the growth would be inhibited due to lower photosynthetic rates and higher respiratory rates [6, 8]. So investigating how photosynthesis and respiration in alpine plants from these regions will respond to high temperature stress is crucial.

However, some alpine species appear unable to respond negatively to high temperatures due to thermal acclimation, which maintains a certain rate of photosynthesis and respiration at the variable growth conditions [6, 9–12]. Thermal acclimation has been shown to optimise carbon gain in three ways: shifting the thermal optimum for net photosynthesis (T_opt_) to improve photosynthetic capacity [4, 10, 13, 14]; altering the quotient of respiration rates given a 10°C change in temperature (Q_10_) (termed type I acclimation); and/or shifting the base respiration rates at a reference temperature (the elevation) of the temperature-response curves for respiration (type II acclimation) [5, 11, 15–18]. Hence temperature acclimation may be a critical factor in predicting how high altitude plant fitness will be affected by high temperatures [10, 17].

Variations in thermal acclimation of photosynthesis and respiration among plants may contribute to change in leaf structure and biochemistry [6, 14, 19, 20]. Leaves with high dry mass per unit area (LMA) tend to have lower photosynthetic rates and higher respiratory rates [21]. As an important component of photosynthetic and respiratory apparatus, high leaf nitrogen content per unit area (N_area_), and nitrogen use per leaf, indicates large investment in photosynthetic and respiratory processes [19, 22]. Therefore, when plants grown in high temperature conditions, the compensatory mechanism of changes in LMA, N_area_ and leaf nitrogen use efficiency may be associated with photosynthetic and respiratory acclimation [5, 23–27].

Members of the genus *Picea* (spruces) are sciophilous trees that form a dominant component in many alpine and boreal evergreen forests [28, 29]. The species are genetically similar to one another, and introgression occurs [30–32]. Increased temperatures have been shown to reduce net carbon uptake in two *Picea* species [8, 25, 26] and if this applies across the genus, then climate change could have a profound effect upon the carbon balance of boreal forests. Therefore, the current study examined temperature acclimation and carbon balance in seedlings of four *Picea* taxa, which originated from two distinguished altitudinal regions. We selected two lower altitude species from the southern limit of boreal coniferous forests (*P*. *koraiensis* and *P*. *meyeri*), and two alpine congeneric species, *P*. *likiangensis* vars. *rubescens* and *linzhiensis*, distribution on the Qinghai-Tibet Plateau, which constitutes the highest alpine treelines in the northern hemisphere [33]. In these regions, plants are more sensitive to temperature than other regions [34, 35]. Therefore, the objectives of our study were to examine the effect of high growth temperature on the growth, photosynthesis and respiration rates of *Picea* taxa. We also determined whether the degree of acclimation of photosynthesis and respiration would differ between two *Picea* groups. Our hypothesis was that *Picea* species were more prone to high temperature stress; and lowland taxa showed higher thermal acclimation than alpine taxa, because of lower elevated temperature compared with higher mean growth season temperature (T_growth_) of lowland taxa in higher growing temperature.

## Materials and Methods

### Ethics Statement

No specific permits were required for the described field studies. Because four *Picea* taxa were not endangered or protected species, and collecting seeds of each Picea taxa from its distribution range did not involve any National Nature Reserve in China. Additionally, this study was conducted at Plant Germplasm Repository, Lanzhou University, an experimental base for our group (Division of molecular Ecology in State Key Laboratory of Grassland Agro-Ecosystems).

### Plant materials and growth conditions

*Picea likiangensis* var. *rubescens* and *P*. *likiangensis* var. *linzhiensis* are distributed between 2900 m and 4200 m a.s.l on the Qinghai-Tibet Plateau, whereas *P*. *koraiensis* and *P*. *meyeri* occur from 400 m (Northeast China) to 2700 m (North China) a.s.l. All seeds of each taxon were collected from the centre of its distribution range, and then were germinated in a common garden plantation in the Plant Germplasm Repository, Lanzhou University, China (35°56'37" N, 104°09'05" E, Alt: 1750 m). All seedlings grown in this plantation though three years to acclimate to the fluctuation of temperature in the growing season (May—August) from 25.8°C (day) to 7.7°C (night). In mid-January 2009, prior to the beginning of the fourth growing season, all seedlings were planted in pots (upper inner diameter: 24 cm, basal diameter: 16 cm, height: 17 cm). Each pot was filled with a homogeneous mixture comprising equal volumes of peat soil and perlite. On 15 June 2009 when new twigs of each spruce were only start growing, eighteen pots of each taxon, accordance of the same height were selected and divided into two growth temperature conditions. Nine pots of each spruce were grown at 25 ± (S.D.) 0.56°C (day) and 15 ± (S.D.) 0.45°C (night) in a growth chamber [moderate temperature (MT)], whereas other half of the pots were moved to an adjacent 35 ± (S.D.) 0.65°C (day) and 25 ± (S.D.) 0.51°C (night) chamber [high temperature (HT)]. In all chambers, light intensity during the day time was ~ 300 μmol photons m^-2^ s^-1^, and relative humidity was 50 ± 5%. The growing period was 15 June to 15 October 2009; day and night lengths were 12 h each, and each pot received ample watering to avoid any effect on the results from water deficit.

Non-destructive growth measurements were carried out at the beginning (15 June 2009; t_1_) and the end (15 October; t_2_) of temperature treatments. Stem height (H) and basal stem diameter (D) were measured on all samples of each temperature treatment. Stem diameter measurements were made using a digital calliper at 0 cm above the soil. A steel tape was used for stem height measurements. Using basal diameter (D) and height (H) measurements, the stem volume index was calculated as D^2^H, which is usually strongly correlated with above-ground biomass [36]. Therefore, the approximated biomass increment during the experimental period was estimated by ΔD^2^H, which was calculated by $D_{t2}^{2}H_{t2}-D_{t1}^{2}H_{t1}$.

### Gas exchange and LMA measurements

Leaf-level gas exchange measurements were made on fully expanded current-year needles with a portable open-path gas exchange system with CO_2_ control (Li-6400, LI-COR Biosciences, Inc.) using a Li-6400-07 Needle Chamber, with an external light source (light levels: ~ 800 μmol m^-2^ s^-1^). At least five random seedlings for each treatment per species were measured on the end period. During the measurements, the CO_2_ concentration was maintained at the atmosphere levels (~ 380 μmol·mol^-1^). Firstly, net photosynthetic rates (P_growth_) and dark respiratory rates (R_growth_) were measured at the growth temperature in HT and MT leaves on 15 October. Secondly, six-point temperature response functions of photosynthetic and respiratory rates (15–40°C in 5°C intervals high to low) were determined on next four days (one species per day). The respiratory rates were measured after a short dark period (between 5 and 10 min) to ensure that photosynthetic activity had ceased. Measurement temperatures were obtained by changing the air temperature of growth chamber, and micro-changing by Li-6400 temperature control system. Between each step change in temperature, we at least waited 30 min for them to stabilize before measuring the samples in random order. After the measurements of gas exchange, needles were harvested and leaf area (LA) per sample was determined using a LI-3100A portable area meter (LI-COR Biosciences, Inc.). Each sample was then oven- dried for 72 h at 65°C, its dry mass determined and LMA calculated. All gas exchange parameters were reported on a needle dry mass basis, which were converted by their corresponding LMA.

### Leaf N nitrogen concentrations

We measured the concentration of nitrogen (N) using leaf samples from the gas exchange measurements. These dry samples were finely ground with mortar and pestle, and then leaf nitrogen content per unit mass was determined using a CHN analyzer (Vario EL, Elementar, Germany) at the Analytical Testing Centre, Lanzhou University, China. Leaf N content per unit area (N_area_) was calculated by multiplying leaf nitrogen content per unit dry mass by the LMA. The nitrogen use efficiency of photosynthesis (PNUE) at each growing temperature was calculated by dividing photosynthetic rates (P_growth_) per unit area by N_area_.

### Data analysis

The photosynthesis data from temperature response curves were used to determine the temperature dependence. To estimate optimum temperature for photosynthesis (T_opt_) and the photosynthetic rate at the optimum temperature (P_opt_), photosynthesis-temperature response curves were fitted with a quadratic equation [4, 37]:

$$P_{T}=aT^{2}+bT+c$$ (1)

Where P_T_ represents the mean net photosynthetic rate at temperature T in°C; a, b and c are fitting parameters for the quadratic curve. T_opt_ was the x-value corresponding to the peak of the quadratic curve which was calculated using the equation $-\frac{b}{2a}$. Likewise, P_opt_ was the peak y-value of the equation $\frac{4ac-b^{2}}{4a}$.

Respiratory temperature response curves were analysed as previously described [12], where respiration rate (R) at a given temperature in given by:

$$R=R_{15}Q_{10}^{\frac{T-15}{10}}$$ (2)

Where R_15_ is the estimated specific base respiration rate at the reference temperature of 15°C, T is foliage temperature and Q_10_ is a parameter, describing the proportional change in respiration with a 10°C increase in temperature.

Quantifying the degree of temperature acclimation of each parameter for each Picea taxon and geographical groups was calculated as the ratio of P_growth_ and R_growth_ measured at 35°C in HT leaves to that measured at 25°C in MT leaves (HT/MT). As an index for the degree of the acclimation, a ratio that is close to 1.0 indicates that the temperature acclimation is high [6, 19].

### Statistical analysis

The parameters from temperature response curves for net photosynthesis were estimated by fitting a second-order polynomial (Eq 1) to each curve. And individual respiratory temperature response curves were fitted using nonlinear regression (Eq 2). If the coefficient of determination (*R* ^2^) for one curve was less than 0.8, this curve was removed. Hence, there were 3–5 replicates in every growth condition for each species to use for nonlinear regression and statistical analysis in *Picea* taxon level. The significance of all differences in traits between *Picea* taxa for each treatment, or between treatments for each taxon at the 5% level were determined by one-way analyses of variance (ANOVA) and Tukey test for multiple comparisons. Meanwhile, we integrated all individuals (n = 6–10) from the same group to evaluate differences between alpine and lowland groups. Differences in traits between groups for each treatment, or between treatments for each group at the 5% level were tested with one-way ANOVA. All regressions and statistical analysis were performed with SPSS 16.0 (SPSS Inc., Chicago, IL, USA).

## Results

### Temperature effects on the gas exchange characteristics and plant growth

**Fig 1:**
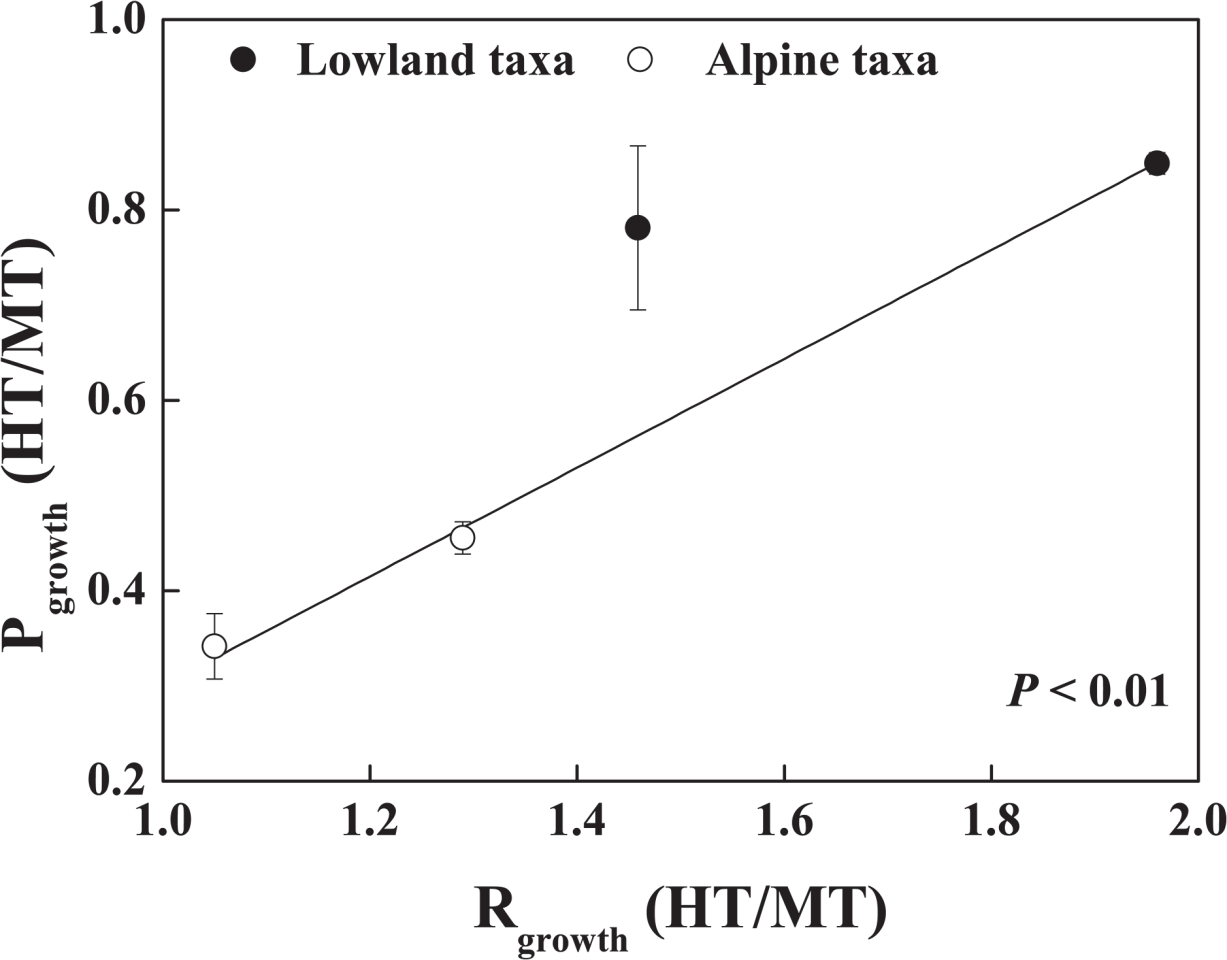
Relationships of thermal acclimation between respiration and photosynthetic rates among four *Picea* taxa. The ratio for degree of the acclimation, P_growth_ (MT/HT) and R_growth_ (MT/HT), is close to 1.0 indicates that the temperature acclimation is high. The solid line represented the regression line, and significance of regression (*P*) were shown. Values are the mean and SE; n = 3 ~ 5.

P_growth_ measured at 25°C (MT) was higher in the alpine *Picea* taxa than in the lowland taxa (Table 1), and significantly different across *Picea* taxa, such that P_growth_ for *P*. *likiangensis* var. *linzhiensis* > P_growth_ for *P*. *likiangensis* var. *rubescens* > P_growth_ for *P*. *meyeri* > P_growth_ for *P*. *koraiensis* MT leaves (S1 Table). Comparing temperature treatments, P_growth_ was significantly lower at 35°C (HT) leaves than at 25°C (MT) leaves for two altitude groups and all taxa except *P*. *meyeri* (Table 1 and S1 Table). After 35°C stress, P_growth_ for the lowland *Picea* taxa showed significantly higher than that for the alpine taxa (Table 1). Comparing P_growth_ at HT leaves among taxa, the value for *P*. *meyeri* was significantly higher than that for either variety of *P*. *likiangensis*; *P*. *koraiensis* was intermediate (S1 Table). Based on this, the index of P_growth_ (HT/MT) was higher at the lowland taxa (Table 1 and Fig 1), suggesting the lowland *Picea* taxa exhibited greater homeostasis than the alpine taxa in response to high temperature stress.

**Table 1:** Temperature acclimation of photosynthesis and respiration, and the approximated biomass increment (Δ*D* ^2^ *H*) in the lowland and alpine *Picea* taxa.

	*P*_growth_ (nmol CO_2_ g^-1^ s^-1^)		Ratio	*R*_growth_ (nmol CO_2_ g^-1^ s^-1^)		Ratio	Δ*D* ^2^ *H* (cm^3^)	
	MT	HT	(acclimation)	MT	HT	(acclimation)	MT	HT
Low altitude taxa	12.73 ± 0.70 aA	10.33 ± 0.62 aB	0.82±0.04 a	5.39 ± 0.33 aA	8.87 ± 0.69 aB	1.66 ± 0.11 a	5.77 ± 0.08 aA	3.52 ± 0.08 aB
High altitude taxa	18.98 ± 0.31 bA	7.52 ± 0.48 bB	0.40±0.03 b	6.61 ± 0.27 bA	7.75 ± 0.28 aB	1.20 ± 0.06 b	6.83 ± 0.14 bA	3.44 ± 0.11 aB

Notes: Each value represents mean ± SE. Letters after SE values distinguish between statistically separable (*P* < 0.05) values for different temperature treatment (A, B) and different geographical locations in same temperature treatment (a, b). n = 6 ~ 10.

R_growth_ measured at MT leaves was significantly higher in the alpine taxa than the lowland taxa, highest in *P*. *likiangensis* var. *linzhiensis* in particular (Table 1 and S1 Table). For all taxa except *P*. *likiangensis* var. *linzhiensis*, R_growth_ significantly increased with increasing growth temperature in both altitude groups (Table 1 and S1 Table). However, both altitude groups exhibited similar R_growth_ in HT leaves. Among taxa, *P*. *koraiensis* had higher R_growth_ at 35°C than any other (S1 Table); ΔR_growth_ (R_growth_HT—R_growth_MT) also varied, being highest in *P*. *koraiensis* and lowest in *P*. *likiangensis* var. *linzhiensis* (S1 Table). So the index of R_growth_ (HT/MT) for the lowland taxa (1.66 ± 0.11) was significantly higher than that for the alpine taxa (1.20 ± 0.06; Table 1 and Fig 1). Hence, there was higher homeostasis of respiration for the alpine taxa in response to high temperature stress. Meanwhile, the R_growth_ (HT/MT) was significantly related to P_growth_ (HT/MT) (Fig 1), reflecting that homeostasis of respiration and photosynthesis was interdependent.

Overall growth over four months, the approximated biomass increment (ΔD^2^H) was higher at 25°C for the alpine taxa (*P*. *likiangensis* varieties) than for the lowland taxa (Table 1 and S1 Table). In all taxa, ΔD^2^H at 35°C was significantly lower than at 25°C, with the difference larger for two *P*. *likiangensis* varieties (the alpine taxa).

### High temperature adjustments in temperature-response parameters of photosynthesis and respiration

**Fig 2:**
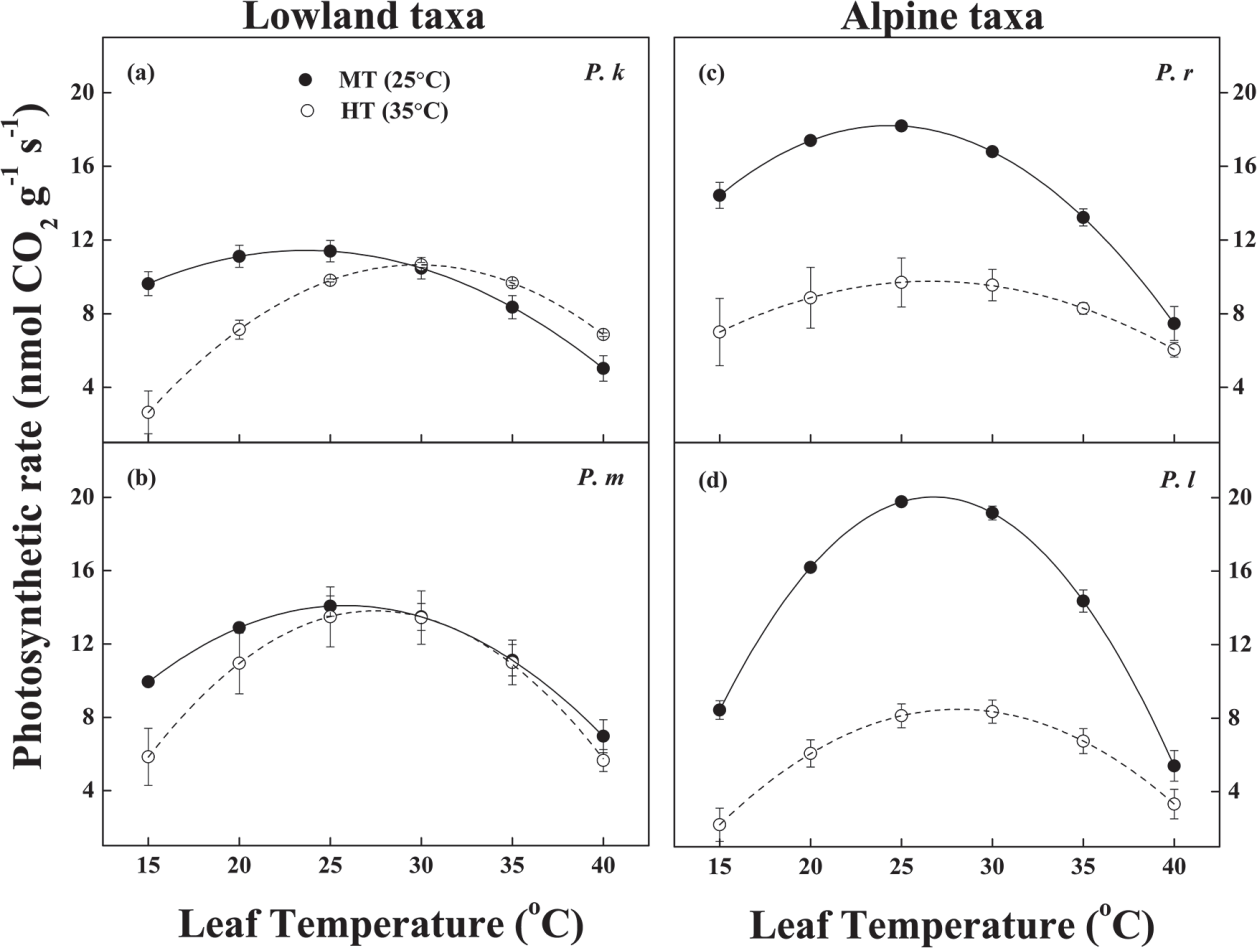
Relationships between leaf photosynthetic rate and temperature for four *Picea* taxa under two temperature treatments. (a) *Picea koraiensis* (*P*. *k*), (b) *P*. *meyeri* (*P*. *m*), (c) *P*. *likiangensis* var. *rubescens* (*P*. *r*), and (d) *P*. *likiangensis* var. *linzhiensis* (*P*. *l*). Each value is presented by mean and SE, n = 3 ~ 5.

High temperature stress treatment resulted in a shift in the temperature response curves for net photosynthesis (Fig 2), such that leaves in HT conditions had higher T_opt_ than that in MT conditions, except for *P*. *meyeri* (Table 2 and S1 Table). T_opt_ differed significantly between taxa for MT leaves but not for HT leaves. But there was no significant differences in T_opt_ between alpine and lowland taxa for HT or MT leaves (Table 2).

**Table 2:** Effects of growth temperature and geographical locations on several measured indicators concerned with the temperature acclimation of photosynthesis and respiration.

Variables		Low altitude taxa	High altitude taxa
*T*_opt_ (°C)	MT	24.71 ± 0.54 aA	25.46 ± 0.54 aA
	HT	28.59 ± 0.54 bA	28.23 ± 0.70 bA
*P*_opt_ (nmol CO_2_ g^-1^ s^-1^)	MT	12.78 ± 0.71 aA	19.15 ± 0.37 aB
	HT	12.23 ± 0.99 aA	9.26 ± 0.69 bB
*PNUE* (nmol CO_2_ mg^-1^ s^-1^)	MT	0.74 ± 0.03 aA	1.27 ± 0.11 aB
	HT	0.53 ± 0.03 bA	0.49 ± 0.05 bA
*R*_15_ (nmol CO_2_ g^-1^ s^-1^)	MT	2.47 ± 0.19 aA	3.28 ± 0.20 aB
	HT	2.14 ± 0.10 aA	2.77 ± 0.18 aB
*Q*_10_	MT	2.23 ± 0.11 aA	1.98 ± 0.04 aA
	HT	2.04 ± 0.10 aA	1.74 ± 0.02 bB
*LMA* (g m^-2^)	MT	235.22 ± 4.59 aA	212.08 ± 11.67 aA
	HT	229.24 ± 7.85 aA	181.58 ± 7.88 bB
*N*_area_ (g m^-2^)	MT	4.05 ± 0.09 aA	3.53 ± 0.43 aA
	HT	4.47 ± 0.19 aA	3.22 ± 0.49 aA

Notes: Each value represents mean ± SE. Letters after SE values distinguish between statistically separable (*P* < 0.05) values for different temperature treatment (a, b) and different geographical locations in same temperature treatment (A, B). n = 6 ~ 10.

Comparing temperature treatments, high growth temperature decreased P_opt_, but this effect was significantly pronounced in the alpine taxa, *P*. *likiangensis* var. *linzhiensis* and *P*. *likiangensis* var. *rubescens* (Table 2, Fig 2 and S1 Table). For MT leaves, P_opt_ was higher in the alpine taxa than the lowland taxa, such that P_opt_ for *P*. *likiangensis* var. *linzhiensis* > P_opt_ for *P*. *likiangensis* var. *rubescens* > P_opt_ for *P*. *meyeri* > P_opt_ for *P*. *koraiensis* leaves. This pattern was reversed after high temperature stress treatments (Table 2). *P*. *meyeri* had a higher value than the two alpine taxa (S1 Table).

**Fig 3:**
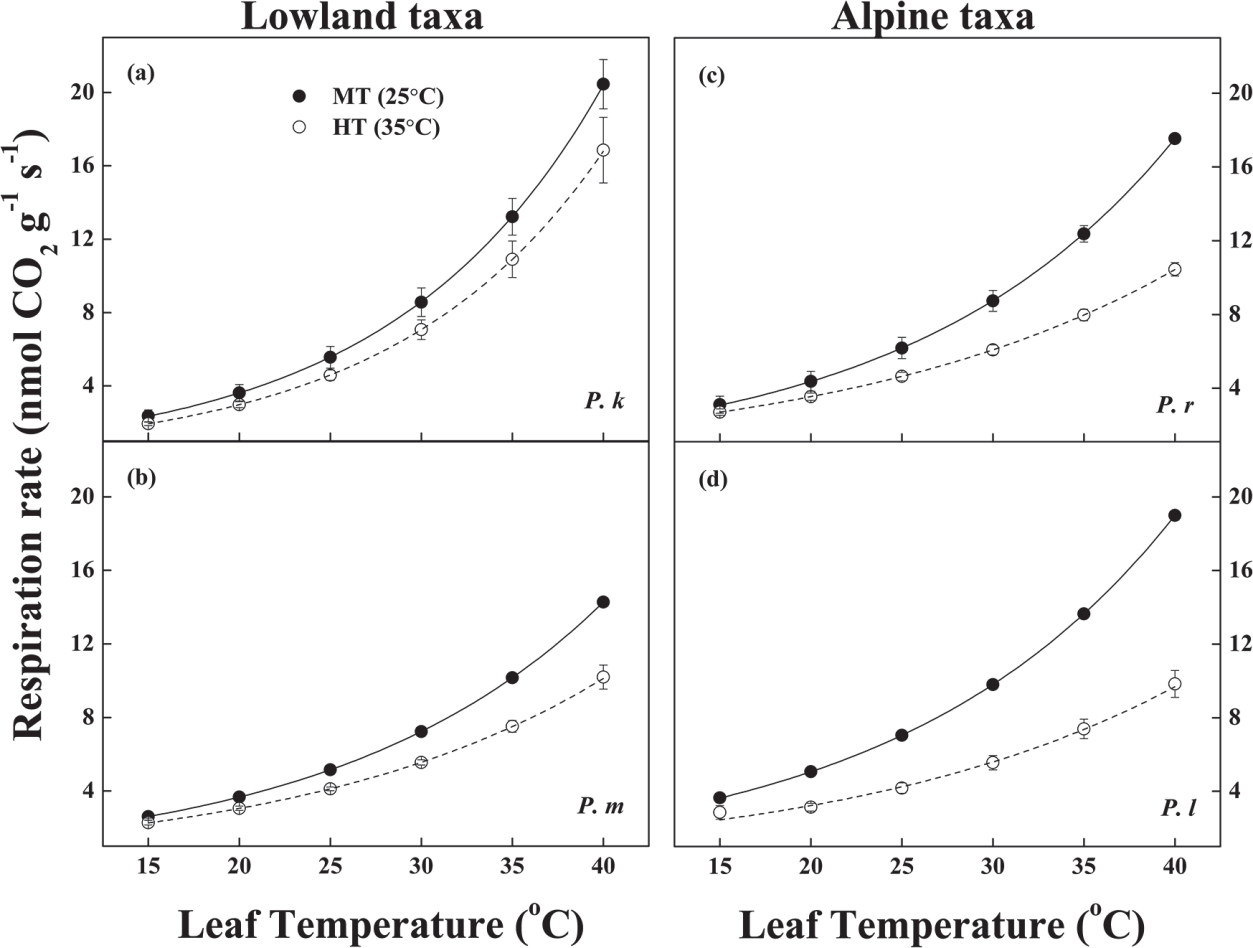
Temperature response curves of the respiratory rate in four *Picea* taxa under two temperature treatments. (a) *Picea koraiensis*, (b) *P*. *meyeri*, (c) *P*. *likiangensis* var. *rubescens*, and (d) *P*. *likiangensis* var. *linzhiensis*. See Fig 2 for species abbreviations. Each value is presented by mean and SE, n = 3 ~ 5.

For the respiration of lowland taxa (*P*. *koraiensis* and *P*. *meyeri*), the temperature-response functions displayed the constant values in the elevation (R_15_) and slope (Q_10_, 15–40°C) of the curves both in MT and HT conditions (Table 2, Fig 3A and 3B and S1 Table). By contrast, high temperature stress treatment only adjusted in downward slopes (Q_10_, 15–40°C) in the alpine taxa (*P*. *likiangensis* var. *rubescens* and *P*. *likiangensis* var. *linzhiensis*) (Table 2, Fig 3C and 3D and S1 Table). Furthermore, R_15_ was significantly lower for lowland taxa, whereas Q_10_ were higher for lowland taxa, irrespective of the growth temperature (Table 2).

### Effects of high temperature on leaf construction

As parameters of the leaf construction, the values of LMA and N_area_ exhibited few significant differences among taxa. In HT leaves, LM*A* was lower in *P*. *likiangensis* var. *linzhiensis* than in other taxa, and in the alpine taxa relative to the lowland taxa (Table 2 and S1 Table). Furthermore, between the alpine (but not the lowland) taxa, LMA was higher in MT than in HT leaves. The only notable differences for N_area_ in HT leaves was that *P*. *meyeri* had higher N_area_ than other taxa (S1 Table), and the values of N_area_ was lower in the alpine taxa relative to the lowland taxa (Table 2). Meanwhile, the nitrogen use efficiency of photosynthesis (PNUE) was lower in HT leaves than that in MT leaves, and higher in the alpine taxa relative to the lowland taxa at MT (Table 2 and S1 Table).

## Discussion

Temperature is one of the most important factors affecting plant growth and distribution [35, 38, 39]. Here, we used a combination of gas exchange parameters and growth characteristics of four *Picea* taxa grown at two different temperatures, and detected two clear patterns in their long-term responses to high temperature stress: first, the alpine taxa showed higher respiration acclimation, and the lowland taxa exhibited greater photosynthetic acclimation; and second, high temperature inhibited growth in *Picea* taxa, especially the larger reduction in high altitude taxa.

### Photosynthetic acclimation of *Picea* seedlings to high temperature

Our results showed that the alpine taxa had higher P_growth_ than the lowland taxa in MT [25°C /15°C (day/night temperature)] (Table 1 and S1 Table), similar to patterns observed in *Crepis pygmaea* and *Isatis apennina* [40]. Two altitude groups had lowered photosynthetic rates (P_growth_) at HT [35°C /15°C (day/night temperature)], relative to MT (Table 1), indicating that photosynthetic acclimation at high temperature stress was not complete in any of these species [10]. Previous studies had reported an upward shift in T_opt_ to acclimate to a higher temperature [4, 13, 41]; in *Picea* there was an upward shift in T_opt_ of 3.32°C (from 25.09°C to 28.41°C in average; Table 2 and S1 Table) to weaken to the high temperature stress.

There was disagreement over the relative importance of climate of origin, and temperature treatment, for causing shifts in T_opt_ [41–44]. Our data showed a similar effect to that found by [4]: inherent differences between species, such as the climate in the range or provenance of the species had much less effect on T_opt_ than the temperature they were grown at *in vitro* (Table 2 and S1 Table). Likewise, previous findings that elevated temperatures above T_opt_, led to reductions in P_opt_ [14, 37, 45] and P_growth_ [8], were supported: both quantities decreased above T_opt_ in our *Picea* species, especially the two alpine taxa (*P*. *likiangensis* vars. *rubescens* and *linzhiensis*; Table 2 and S1 Table).

Variations in P_opt_ may also be related to differences in leaf constructions and biochemical processes [25, 26, 46–48]. Work on leaf economics spectrum (LES) had already suggested that leaf photosynthesis was closely related with leaf N_area_ and LMA [21]. Increased N_area_ and decreased LMA can both be compensatory mechanisms for the deleterious effects of high temperature [23, 24, 27]. In our study, only slight deceased values of LMA between temperature treatments for the alpine taxa were suggested high temperature stress changed in the leaf anatomy and density for the alpine taxa (Table 2 and S1 Table). However, changed in LMA was unlikely to explain temperature treatment differences in P_opt_. Further, the constant values of N_area_, but reduction in PNUE were suggest high temperature stress inhibited the photosynthetic enzymatically catalysed reactions and the electron transport capacity [27, 49, 50] Meanwhile, these results were suggested nitrogen use was more important for photosynthetic acclimation than nitrogen content [19].

Overall, *Picea* taxa varied in the extent to which P_growth_ decreases at higher temperatures, indicating that the thermal acclimation of photosynthesis was species-specific, and might be closely related to the natural distribution of taxa [10, 35, 41], because of lower elevated temperature compared with higher mean growth season temperature of the lowland taxa in higher growing temperature.

### Respiration acclimation of *Picea* seedlings to high temperature

As we expected, the alpine taxa had higher base respiratory rates (R_15_) than the lowland taxa (Tables 1 and 2 and S1 Table), because of the lower temperature of their growth seasons (T_growth_) in high altitudes. [18] also found that the base respiratory rate at 5°C was higher in cooler periods for *Pinus banksiana*. At high temperature stress (35°C), the R_growth_ for all taxa were increased (Table 1 and S1 Table), reflected increased demand for energy to maintenance [12]. However, different altitude taxa showed varied R_growth_, caused by different respiratory acclimation. Thermal acclimation of respiration resulted in an alteration in the shape or elevation of the temperature-response curve as plants were grown in warmer temperatures [5, 12]. And, Q_10_ and R_15_ derived from curve-fitting procedures were not strictly independent [18]. In our study, only significantly downward adjustment in Q_10_ (15–40°C) with no changes in R_15_ between MT and HT treatment in the alpine taxa (*P*. *likiangensis* var. *rubescens* and *P*. *likiangensis* var. *linzhiensis*) were consistent with a Type I acclimation [5, 51], unlike the lowland *Picea* taxa. Meanwhile, the variation in thermal acclimation of respiration was underpin by the larger changed in Q_10_ with high temperature stress than slight adjustment of R_15_ (Table 2, Fig 3 and S1 Table).

A significant downshift in Q_10_ at higher temperatures for the alpine *Picea* taxa indicates that the alpine taxa had higher ability of adjustment in the temperature sensitivity of the temperature-response function at high temperature stress. These larger reductions of Q_10_ resulted in limited R_growth_ of the alpine taxa via regulatory changes in respiratory enzymes, in particular, lack of substrate availability and degree of adenylate at long-term high temperature stress [5, 51]. Because long-term high temperature stress restricted their photosynthetic rates (Table 1 and S1 Table) [12]. This reflected the interdependence of respiration and photosynthesis. Meanwhile, respiration equally might be limiting photosynthesis, because photosynthesis likewise depends on respiration for a range of compounds (e.g. ATP) [17; 46]. Therefore, the thermal acclimation of respiration would be related to photosynthetic acclimated to temperatures [19]. As a result, different respiratory acclimation were highly correlated with the photosynthetic acclimation (Fig 1). However, there were smaller declines in Q_10_, and higher uplifts in R_growth_ in the lowland taxa (Table 2 and S1 Table), indicating a lower degree of respiration acclimation in the lowland taxa (Table 1 and Fig 1).

### *Picea* seedlings of diverse climatic origin differ in the acclimation of photosynthesis and respiration

Growth seasons were warmer at lower altitudes, and plants had developed various mechanisms to enhance their tolerance to higher temperatures stress via adjustments of physiological and morphological characteristics [4, 10]. Greater tolerance of higher temperatures stress in the lowland taxa was indicated by higher LMA and N_area_, and a smaller shift in PNUE between moderate and high temperatures. This meant more carbon partitioning to preventive architecture, more proteins and higher stability of photosynthetic enzymatically catalysed reactions and membrane processes. Therefore, there was a greater degree of photosynthesis acclimation in the lowland taxa than in the alpine taxa (Table 1 and Fig 1), and photosynthetic apparatus for the lowland taxa was more tolerant of high temperature.

In the lowland taxa, relative to the alpine taxa, R_growth_ at HT was higher but acclimation of respiration was lower. This was caused by the more stable photosynthetic process of the lowland taxa (Fig 1), which need more energy for maintenance to protein turnover and membranes at high temperature stress [12]. Meanwhile, higher *R*_growth_ prevented chloroplast over-redox at high temperature [12]. Generalisations about the effects of altitude on acclimation potential were difficult because of contrasting results from earlier studies [11, 17]; however, high acclimation of respiration was often associated with changing in *LMA* [12, 16]. Our results for *P*. *likiangensis var*. *linzhiensis* supported this hypothesis. However, leaves of slow-growing conifers like *Picea* may had low potential acclimation than those of fast-growing species [17].

### Inhibition of growth when *Picea* taxa are exposed to high temperature

Trees growing naturally in cooler regions were often assumed to be temperature limited [6]; while moderate warmer temperatures would enhance growth [52]. If temperature rises beyond the thermal optimum of growth, the resulting imbalance of photosynthesis and respiration may cause inhibition of growth [8, 25, 26]. We found that biomass accumulation (ΔD^2^H) in all four *Picea* taxa was reduced at HT (35°C daytime temperature), indicating inhibition of growth [25, 26]. Various *Picea* taxa, including boreal species, had been shown to have acclimated by shifting *T*_opt_ when temperature changes (either in the laboratory or in the field) [4, 13, 41, 51]. This can involve interactions between the physiological and morphological characteristics, such as changes in photosynthetic and respiratory capacities and leaf structure [10, 12, 16, 22, 53]. However, the small shifts in *T*_opt_ (3.3°C) observed here were not enough to compensate for deleterious effects of long-term exposure to high temperature stress, confirming that daytime temperature stress of 35°C exceed *Picea* species’ capacity to adjust their thermal optimum [8].

## Conclusions

Overall, our results suggested that stress imposed by high growth temperature reduced the growth of *Picea* seedlings, causing decreased photosynthesis and increased respiration. Furthermore, we found that the lowland taxa (*P*. *koraiensis* and *P*. *meyeri*) exhibited higher photosynthetic acclimation and lower respiratory acclimation than the alpine taxa (*P*. *likiangensis* vars. *rubescens* and *linzhiensis*). Photosynthetic acclimation had been considered to be related to changes in N_area_ and LMA. However, the variations in photosynthetic acclimation were mainly determined by differences in PNUE. On the other hand, the extent of respiratory acclimation was considered to interact with photosynthetic rates. Thus, seedlings of the alpine taxa (*P*. *likiangensis* var. *rubescens* and *P*. *likiangensis var*. *linzhiensis*) were more susceptible to high temperature than the lowland taxa (*P*. *koraiensis* and *P*. *meyeri*).

## Supporting Information

S1 Table Comparison of all measured indicators between 25°C (MT) and 35°C (HT) treatments in each *Picea* taxon. (DOC)
