# Supplementary material for: Higher Thermal Acclimation Potential of Respiration but Not Photosynthesis in Two Alpine Picea Taxa in Contrast to Two Lowland Congeners
Source: PLoS One. 2015 Apr 13;10(4):e0123248. doi: 10.1371/journal.pone.0123248 (PMC4395334; doi:10.1371/journal.pone.0123248)
Supplement: S1 Table — (DOC) [file pone.0123248.s001.doc]

**S1 Table.** Comparison of all measured indicators between 25 °C (MT) and 35 °C (HT) treatments in each *Picea* taxon.

| Variables |  | | Low altitude taxa | |  | High altitude taxa | |
| --- | --- | --- | --- | --- | --- | --- | --- |
|  |  |  | *P. koraiensis* | *P. meyeri* |  | *P. likiangensis* var*. rubescens* | *P. likiangensis* var. *linzhiensis* |
| *P*_growth_ | | MT | 11.39 ± 0.58 aA | 14.07 ± 0.55 aB |  | 18.19 ± 0.14 aC | 19.77 ± 0.14 aD |
|  | | HT | 9.67 ± 0.13 bAB | 10.99 ± 1.21 aA |  | 8.29 ± 0.31 bBC | 6.75 ± 0.68 bC |
| *T*_opt_ | | MT | 23.66 ± 0.06 aA | 25.76 ± 0.60 aB |  | 24.15 ± 0.35 aA | 26.77 ± 0.30 aB |
|  | | HT | 29.70 ± 0.32 bA | 27.48 ± 0.37 aA |  | 28.35 ± 1.56 bA | 28.11 ± 0.03 bA |
| *P*_opt_ | | MT | 11.44 ± 0.58 aA | 14.13 ± 0.59 aB |  | 18.23 ± 0.13 aC | 20.06 ± 0.22 aD |
|  | | HT | 10.67 ± 0.13 aAB | 13.80 ± 1.57 aA |  | 10.03 ± 1.16 bB | 8.48 ± 0.63 bB |
| *PNUE* | | MT | 0.68 ± 0.03 aA | 0.79 ± 0.03 aB |  | 0.97 ± 0.01 aC | 1.58 ± 0.01 aD |
|  | | HT | 0.52 ± 0.01 bAB | 0.53 ± 0.06 bAB |  | 0.39 ± 0.01 bB | 0.59 ± 0.06 bA |
| *R*_growth_ | | MT | 5.56 ± 0.60 aA | 5.15 ± 0.13 aA |  | 6.18 ± 0.41 aAB | 7.05 ± 0.20aB |
|  | | HT | 10.91 ± 0.99 bA | 7.51 ± 0.32 bB |  | 7.97 ± 0.31 bB | 7.39 ± 0.53 aB |
| *R*_15_ | | MT | 2.37 ± 0.34 aA | 2.61 ± 0.09 aAB |  | 3.11 ± 0.33 aAB | 3.46 ± 0.25 aB |
|  | | HT | 1.94 ± 0.10 aA | 2.28 ± 0.13 aAB |  | 2.71 ± 0.17 aB | 2.85 ± 0.37 aB |
| *Q*_10_ | | MT | 2.42 ± 0.13 aA | 1.97 ± 0.01 aB |  | 2.02 ± 0.08 aB | 1.94 ± 0.03 aB |
|  | | HT | 2.37 ± 0.09 aA | 1.83 ± 0.08 aB |  | 1.72 ± 0.03 bB | 1.77 ± 0.01 bB |
| Δ*D*^2^*H* | | MT | 5.91 ± 0.11 aA | 5.64 ± 0.10 aA |  | 6.99 ± 0.15 aB | 6.67 ± 0.23 aB |
|  | | HT | 3.35 ± 0.08 bA | 3.69 ± 0.08 bB |  | 3.78 ± 0.07 bB | 3.16 ± 0.02 bA |
| *LMA* | | MT | 235.74 ± 4.29 aA | 234.70 ± 9.31 aA |  | 235.42 ± 10.51 aA | 188.73 ± 5.01 aB |
|  | | HT | 222.89 ± 0.68 aA | 233.05 ± 12.76 aA |  | 211.69 ± 0.62 aA | 166.53 ± 3.59 bB |
| *N*_area_ | | MT | 3.97 ± 0.11 aA | 4.16 ± 0.15 aA |  | 4.41 ± 0.14 aA | 2.36 ± 0.22 aB |
|  | | HT | 4.10 ± 0.18 aA | 4.84 ± 0.05 bB |  | 4.52 ± 0.12 aB | 1.92 ± 0.04 aC |

Notes: Each value represents mean ± SE. Letters after SE values distinguish between statistically separable (*P* < 0.05) values for different temperature treatment (a, b) and different species in same temperature treatment (A, B, C, D). n = 3 ~ 5.
